# *Brucella abortus* Infection Elicited Hepatic Stellate Cell-Mediated Fibrosis Through Inflammasome-Dependent IL-1β Production

**DOI:** 10.3389/fimmu.2019.03036

**Published:** 2020-01-21

**Authors:** Paula Constanza Arriola Benitez, Ayelén Ivana Pesce Viglietti, Marco Tulio R. Gomes, Sergio Costa Oliveira, Jorge Fabián Quarleri, Guillermo Hernán Giambartolomei, María Victoria Delpino

**Affiliations:** ^1^Instituto de Inmunología, Genética y Metabolismo (INIGEM), Universidad de Buenos Aires, CONICET, Buenos Aires, Argentina; ^2^Department of Biochemistry and Immunology, Institute of Biological Sciences, Federal University of Minas Gerais, Belo Horizonte, Brazil; ^3^Instituto de Investigaciones Biomédicas en Retrovirus y Sida (INBIRS), Universidad de Buenos Aires, CONICET, Buenos Aires, Argentina

**Keywords:** *Brucella*, inflammasome, fibrosis, hepatic stellate cells, IL-1β

## Abstract

In human brucellosis, the liver is frequently affected. *Brucella abortus* triggers a profibrotic response on hepatic stellate cells (HSCs) characterized by inhibition of MMP-9 with concomitant collagen deposition and TGF-β1 secretion through type 4 secretion system (T4SS). Taking into account that it has been reported that the inflammasome is necessary to induce a fibrotic phenotype in HSC, we hypothesized that *Brucella* infection might create a microenvironment that would promote inflammasome activation with concomitant profibrogenic phenotype in HSCs. *B. abortus* infection induces IL-1β secretion in HSCs in a T4SS-dependent manner. The expression of caspase-1 (Casp-1), absent in melanoma 2 (AIM2), Nod-like receptor (NLR) containing a pyrin domain 3 (NLRP3), and apoptosis-associated speck-like protein containing a CARD (ASC) was increased in *B. abortus*-infected HSC. When infection experiments were performed in the presence of glyburide, a compound that inhibits NLRP3 inflammasome, or A151, a specific AIM2 inhibitor, the secretion of IL-1β was significantly inhibited with respect to uninfected controls. The role of inflammasome activation in the induction of a fibrogenic phenotype in HSCs was determined by performing *B. abortus* infection experiments in the presence of the inhibitors Ac-YVAD-cmk and glyburide. Both inhibitors were able to reverse the effect of *B. abortus* infection on the fibrotic phenotype in HSCs. Finally, the role of inflammasome in fibrosis was corroborated *in vivo* by the reduction of fibrotic patches in liver from *B. abortus*-infected ASC, NLRP, AIM2, and cCasp-1/11 knock-out (KO) mice with respect to infected wild-type mice.

## Introduction

Human brucellosis is a zoonosis that induces a chronic and debilitating disease caused by *Brucella* species that manifests itself with a broad clinical spectrum (1, 2). Liver involvement in human brucellosis is usually documented, given the well-characterized tropism of *Brucella* for the reticuloendothelial system (1, 2). The incidence of liver involvement in active brucellosis has ranged from 5 to 53% or more (2).

Inflammasome activation has been documented in several liver diseases. Accordingly, it has been postulated that the upregulation of IL-1β and IL-18 secretion leads to myofibroblast differentiation with concomitant increase of collagen and TGF-β expression (3). In addition, it was established that inflammasome components are present in hepatic stellate cells (HSCs) and could regulate their function (3). The consequences of activation of inflammasome pathway were also confirmed *in vivo*, demonstrating its key role in liver fibrosis (4).

The activation and release of IL-1β and IL-18 requires two distinct signals. TLR engagement by pathogen or endogenous signal induces the expression of the precursor forms of these cytokines (pro–IL-1β and pro–IL-18), after which NLR-dependent activation of caspase-1 regulates their proteolytic processing and release (5). Activation of inflammasome by *Brucella abortus* infection has been previously demonstrated in bone marrow-derived macrophages and dendritic cells (6, 7). In these cells, *B. abortus* induces the secretion of IL-1β, in a process in which NLRP3 is necessary for activation of ASC inflammasome and the concomitant activation of caspase-1 and maturation and secretion of IL-1β (6, 7). In addition, ASC inflammasomes are also essential for IL-1β secretion induced by *B. abortus* infection in astrocytes and microglia (8). The first signal can be triggered by various pathogen-associated molecular patterns (PAMPs) via TLR activation. In the case of *B. abortus* infection inflammasome activation, the second signal involved the presence of a functional type 4 secretion system (T4SS) and DNA-sensing inflammasome receptor AIM2, in bone marrow-derived macrophages, and Mal/TIRAP and TLR-2 are the main signaling involved in astrocytes and microglia (8).

Previously, we have demonstrated that upon infection of HSCs, *B. abortus* triggers a profibrotic response characterized by inhibition of MMP-9 secretion inducing concomitant collagen deposition and transforming growth factor (TGF)-β1 secretion in a way that involves a functional T4SS and its effectors protein BPE005 (9). Taking into account that inflammasome has been documented to be necessary to induce activation to a fibrotic phenotype of HSCs, we hypothesized that *Brucella* infection might create a microenvironment that would promote inflammasome activation and concomitant profibrogenic phenotype in HSCs. The results of the study are presented here.

## Materials and Methods

### Bacterial Culture

*Brucella abortus* S2308 DsRed-expressing *B. abortus* S2308 or the isogenic *B. abortus virB10* polar mutants were grown overnight in 10 ml of tryptic soy broth (Merck, Buenos Aires, Argentina) with constant agitation at 37°C. Bacteria were harvested and the inocula were prepared as described previously (10).

To obtain heat-killed *B. abortus* (HKBA), bacteria were washed five times for 10 min each in sterile PBS, heat killed at 70°C for 20 min, aliquoted, and stored at −70°C until they were used. The total absence of *B. abortus* viability after heat killing was verified by the absence of bacterial growth on tryptose soy agar.

All live *Brucella* manipulations were performed in biosafety level 3 facilities located at the Instituto de Investigaciones Biomédicas en Retrovirus y SIDA (INBIRS).

### Cell Culture

LX-2 cell line, a spontaneously immortalized human HSC line, was kindly provided by Dr. Scott L. Friedman (Mount Sinai School of Medicine, New York, NY). LX-2 cells were maintained in DMEM (Life Technologies–Invitrogen, Carlsbad, CA, USA) and supplemented with 2 mM L-glutamine, 100 U/ml penicillin, 100 μg/ml streptomycin, and 2% (v/v) fetal bovine serum (FBS; Gibco–Invitrogen, Carlsbad, CA, USA). All cultures were grown at 37°C and 5% CO_2_.

### Cellular Infection

LX-2 cells were seeded in 24-well-plates and infected with *B. abortus* S2308, DsRed-expressing *B. abortus* S2308, or its isogenic mutants at multiplicities of infection (MOI) of 100 and 1000. After the bacterial suspension was dispensed, the plates were centrifuged for 10 min at 2,000 rpm and then incubated for 2 h at 37°C under a 5% CO_2_ atmosphere. Cells were extensively washed with DMEM to remove extracellular bacteria and incubated in medium supplemented with 100 μg/ml gentamicin and 50 μg/ml streptomycin to kill extracellular bacteria. LX-2 cells were harvested at different times to determine cytokine production, MMP secretion, and collagen deposition.

### Neutralization Experiments

Neutralization experiments were performed with 5 μM of Bay 11-7082, an inhibitory compound of the nuclear factor-κB (NF-κB), 50 μM of glybenclamide (glyburide), an inhibitor of the NLRP3 inflammasome, 50 μM of general caspase inhibitor Z-VAD-FMK, or 50 μM of caspase 1 inhibitor Y-VAD-FMK (all inhibitors were from Sigma-Aldrich). The cells were treated for 1 h with each inhibitor before infection.

AIM2 inflammasome complex formation was prevented using 3 μM of A151, a DNA sequence that inhibits in a competitive manner the immunostimulatory DNA. A151 (5-TTAGGGTTAGGGTTAGGGTTAGGG-3) and the control C151 (5-TTCAAATTCAAATTCAAATTCAAA-3) constructs were synthesized with a phosphorothioate backbone. To determine the implication of IL-1β, neutralization experiments were performed by adding 50 ng/ml of ANAKINRA, the inhibitor of IL-1 receptor, and the natural antagonist IL-1Ra (R&D Systems). Recombinant human IL-1β (rIL-1β, R&D Systems) at a concentration of 50 ng/ml was used as a positive control.

### mRNA Preparation and Quantitative PCR

RNA from LX-2 cells was isolated using the Quick-RNA MiniPrepKit (Zymo Research) and 1 μg of RNA was subjected to reverse transcription using Improm-II Reverse Transcriptase (Promega). PCR analysis was performed with StepOne real-time PCR detection system (Life Technology) using SYBR Green as fluorescent DNA binding dye. The primer sets used for amplification were as follows: β-actin sense: 5′-AACAGTCCGCCTAGAAGCAC-3′, β-actin antisense: 5′-CGTTGACATCCGTAAAGACC-3′; NLRP3 sense: 5′-CCACAAGATCGTGAGAAAACCC-3′; NLRP3 antisense: 5′-CGGTCCTATGTGCTCGTCA-3′; IL-1β sense: 5′-AGCTACGAATCTCCGACCAC-3′; IL-1β antisense: 5′-CGTTATCCCATGTGTCGAAGAA-3′; ASC sense: 5′-TGGATGCTCTGTACGGGAAG-3′; ASC antisense: 5′-CCAGGCTGGTGTGAAACTGAA-3′; Capase-1 sense: 5′-TTTCCGCAAGGTTCGATTTTCA-3′ Caspase-1 antisense: 5′-GGCATCTGCGCTCTACCATC-3′; AIM2 sense: 5′-TGGCAAAACGTCTTCAGGAGG-3′; AIM2 antisense: 5′-AGCTTGACTTAGTGGCTTTGG-3′.

The amplification cycle for Caspase-1, ASC, and β-actin was 95°C for 15 s, 58°C for 30 s, and 72°C for 60 s; the amplification cycle for IL-1β, NLRP3, and AIM2 was 95°C for 15 s, 61°C for 30 s, and 72°C for 60 s. All primer sets yielded a single product of the correct size. Relative expression levels were normalized against β-actin.

### Immunofluorescence

LX-2 cells were infected with *B. abortus*, and after 24 h, cells were fixed in 4% paraformaldehyde for 10 min at room temperature, permeabilized with 0.3% Triton X-100 (Roche Diagnostics GmbH, Mannheim, Germany) for 10 min, and blocked with PBS containing 1% BSA for 1 h. Infected cells were stained with mouse anti-ASC (Santa Cruz Biotechnology) diluted in 0.1% PBS–Tween 20 overnight at 4°C. Cells then were incubated with rabbit anti-mouse Alexa Fluor 488 (Molecular Probes, Life Technologies) diluted in 0.1% PBS–Tween for 4 h at room temperature. 4,6-Diamidine-2-phenylindole (DAPI) was used for nuclear staining, and cells were stained for 30 min at room temperature. After washing in PBS, cells were mounted and then analyzed by fluorescence microscopy. Confocal images were analyzed using FV-1000 confocal microscope with an oil immersion Plan Apochromatic 60 × NA1.42 objective (Olympus).

### Zymography

Gelatinase activity was assayed by the method of Hibbs et al. with modifications, as described (11–13). Briefly, a total of 20 μl of cell culture supernatants from infected LX-2 cells cultured in the presence or not of the inhibitors Bay 11-7082, glyburide, Y-VAD-FMK, A151, control C151, and ANAKINRA at the concentrations mentioned above was mixed with 5 μl of 5 × loading buffer [0.25 M Tris (pH 6.8), 50% glycerol, 5% SDS, and bromophenol blue crystals] and loaded onto 10% SDS-PAGE gels containing 1 mg/ml gelatin (Sigma-Aldrich, Buenos Aires, Argentina). After electrophoresis, gels were washed with a solution containing 50 mM Tris–HCl (pH 7.5) and 2.5% Triton X-100 (buffer A) for 30 min and with buffer A added with 5 mM CaCl_2_ and 1 μM ZnCl_2_ for 30 min and were later incubated with buffer A with an additional 10 mM CaCl_2_ and 200 mM NaCl for 48 h at 37°C. This denaturation/renaturation step promotes MMP activity without the proteolytic cleavage of pro-MMP. Gelatin activity was visualized by the staining of the gels with 0.5% Coomassie blue. Unstained bands indicated the presence of gelatinase activity, and their positions in the gel indicate the molecular weights of the enzymes involved.

### Measurement of Cytokine Concentrations

Secretion of TGF-β1 and IL-1β in the supernatants was quantified by ELISA (BD Biosciences).

### Assessment of Collagen Deposition—Sirius Red Staining

Collagen deposition was quantified using Sirius red (Sigma-Aldrich, Argentina SA), a strong anionic dye that binds strongly to collagen molecules (14). Sirius red staining was performed as was described (15). Briefly, Sirius red was dissolved in saturated aqueous picric acid at a concentration of 0.1%. Bouin's fluid (for cell fixation) was prepared by mixing 15 ml saturated aqueous picric acid with 5 ml of 35% formaldehyde and 1 ml of glacial acetic acid. Cell layers were fixed with 1 ml of Bouin's fluid for 1 h. Afterwards, culture plates were washed three times with deionized water. Culture dishes were air dried before adding 1 ml of Sirius red dye reagent. Cells were stained for 18 h with mild shaking. The stained cell layers were extensively washed with 0.01 N hydrochloric acid to remove all unbound dye. The stained material was dissolved in 0.2 ml of 0.1 N sodium hydroxide by shaking for 30 min. The dye solution was transferred to microtiter plates, and OD was measured using a microplate reader (Thermo Scientific) at 550 nm against 0.1 N sodium hydroxide as a blank.

### Lipoproteins and LPS

*Brucella abortus* lipidated outer membrane protein 19 (LOmp19) and unlipidated Omp19 (U-Omp19) were obtained as described (16). Both contained <0.25 endotoxin U/μg of protein as assessed by Limulus Amebocyte Lysates (Associates of Cape Cod). *B. abortus* S2308 LPS and *Escherichia coli* O111k58H2 LPS were provided by I. Moriyon. The synthetic lipohexapeptide (tripalmitoyl *S*-glyceryl-Cys-Ser- Lys4-OH [Pam3Cys]) was purchased from Boehringer Mannheim (Mannheim, Germany).

### DNA From *B. abortus*

*Brucella abortus* DNA was purified using the kit Wizard^®^ Genomic DNA (Promega) following the instructions of the manufacturer. *Brucella* DNA was measured spectrophotometrically. Transient transfections of LX-2 cells with 2 μg/ml of *B. abortus* DNA were carried out using Lipofectamine 2000 (Invitrogen), following the manufacturer's instructions. After the purification step, an aliquot of 100 μg of DNA was treated with DNase I (1 U/mg) DNA (Zymo Research) according to the instructions of the manufacturer.

### Hepatic Fibrosis in a Mouse Model

Mouse strains used in this study included apoptosis-associated speck-like protein containing a CARD (ASC), Nod-like receptor (NLR) containing a pyrin domain 3 (NLRP3), absent in melanoma 2 (AIM2), and caspase-1 (Casp-1)/11 knock-out (KO) mice, as described previously (8), and C57BL/6 wild-type (WT) mice (provided by Federal University of Minas Gerais, Belo Horizonte, Brazil). Six- to eight-week-old mice were infected through the intraperitoneal route with 5 × 10^5^ CFU of *B. abortus* S2308. Mice were born from breeding pairs that were housed under controlled temperature (22 ± 2°C) and artificial light under a 12-h cycle period. Mice were kept under specific pathogen-free conditions in positive-pressure cabinets and provided with sterile food and water *ad libitum*.

All animal procedures were performed according to the rules and standards for the use of laboratory animals of the National Institutes of Health. Animal experiments were approved by the Institutional Committee for the Care and Use of Laboratory Animals (CICUAL, permit number: 287/2015). Histological examination of liver was carried out at week 4 post-infection after routine fixation and paraffin embedding. Five-micrometer-thick sections were cut and stained with Masson's trichrome stain. Masson's trichrome staining was conducted according to the manufacturer's instructions (Sigma-Aldrich). Collagen-positive areas were visualized by light microscopy and quantified using Image Pro-Plus 6.0 software (Media Cybernetics, Inc.).

### Statistical Analysis

Statistical analysis was performed with one-way ANOVA, followed by *post-hoc* Tukey test (a single-step multiple comparison statistical test that finds means that are significantly different from each other) using GraphPad Prism 4.0 software. Data were presented as mean ± SEM.

## Results

### *B. abortus* Infection Induces IL-1β Secretion by LX-2 Cells via T4SS

**Figure 1:**
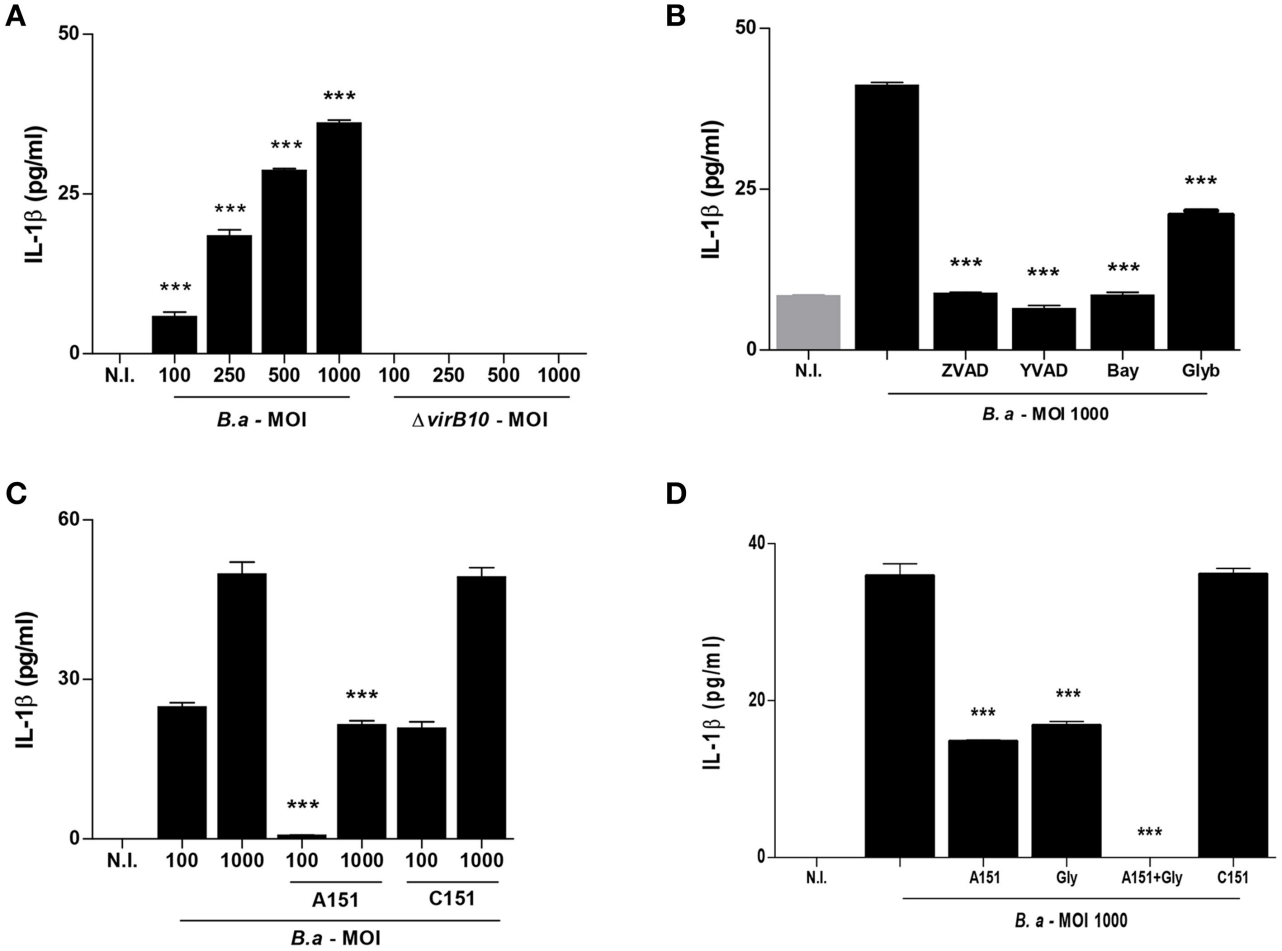
*B. abortus* infection induces IL-1β in a VirB-dependent manner. LX-2 cells were infected with *B. abortus* (*B.a*) and its *vir*B10 isogenic mutant (Δ*vir*B10) at an MOI of 100–1000 and 24 h post-infection; IL-1β secretion was determined by ELISA in culture supernatants **(A)**. Effect of Z-VAD-FMK (ZVAD), Y-VAD-FMK (YVAD), Bay 11-7082 (Bay) and glybenclamide (Glyb) during *B. abortus* infection (MOI 1000) on IL-1β secretion **(B)**. Effect of A151 and the control 151 (C151) during *B. abortus* infection on IL-1β secretion **(C)**. Effect of Glyb plus A151 on IL-1βsecretion during *B. abortus* infection (MOI 1000). Data are given as the means ± SD from at least three individual experiments. ^***^P < 0.001 vs. cells infected with Δ*vir*B10 **(A)** or vs. infected and untreated cells **(B–D)**.

It has been established that inflammasome components are present in HSCs and could regulate their function (3). To determine if *B. abortus* infection induces inflammasome activation, LX-2 cells were infected with *B. abortus* and the secretion of IL-1β was evaluated in culture supernatants by ELISA 24 h post-infection. *B. abortus* infection induces the secretion of IL-1β by LX-2 cells (Figure 1A). The T4SS encoded by *virB* genes was first involved in the capacity of *Brucella* to establish an intracellular replication niche in several cell types (17). In addition, this system has been involved in the induction of inflammatory response during *B. abortus* infection (18) and also in the inflammasome signaling activation (7). Therefore, experiments were conducted to determine if the T4SS could be involved in the secretion of IL-1β induced by *B. abortus* in LX-2 cells. To this end, LX-2 cells were infected with *B. abortus* and its isogenic *B. abortus virB10* mutant, and IL-1β secretion induced by *B. abortus* was dependent on the expression of a functional T4SS, since the levels or IL-1β did not differ significantly between LX-2 cells infected with *B. abortus virB10* mutant and uninfected controls (Figure 1A). Taken together, our results indicated that *B. abortus* infection induces IL-1β secretion in a mechanism that is dependent on the presence of a functional T4SS.

### IL-1β Secretion Induced by *B. abortus* Infection Is Dependent on Caspase-1 and NLRP3

Caspase-1 plays a fundamental role in innate immunity as the protease that activates the pro-inflammatory cytokines pro-IL-1β and pro-IL-18. Caspase-1 itself is activated in different inflammasome complexes; however, activation of the NLRP3 inflammasome has been frequently implicated in the development of fibrosis (19). To determine the role of caspase-1 and NLRP3 in IL-1β secretion by *B. abortus*-infected LX-2 cells, we performed the infection of LX-2 cells in the presence of specific pharmacological inhibitors. Inhibition of caspase-1 using the general caspase inhibitor Z-VAD-FMK or the specific caspase-1 inhibitor Ac-YVAD-cmk completely abrogated the secretion of IL-1β secretion induced by *B. abortus* infection of LX-2 cells (Figure 1B). When infection experiments were performed in the presence of Bay compound that inhibits NFκB, the secretion of IL-1β was significantly inhibited. However, glyburide, a compound that inhibits NLRP3, partially inhibits IL-1β secretion with respect to untreated cells (Figure 1B). Taken together, these results indicated that caspase-1 and NLRP3 are involved in the secretion of IL-1β by *B. abortus*-infected LX-2 cells.

### IL-1β Secretion Induced by *B. abortus* Infection Is Also Dependent on AIM2

AIM2 inflammasome has been previously involved in the induction of IL-1β secretion in a T4SS-dependent manner during *B. abortus* infection of bone marrow derived macrophages and dendritic cells (6, 7). Since the inhibition of NLRP3 did not completely abrogate the secretion of IL-1β in response to *B. abortus* infection, experiments were conducted to determine whether AIM2 inflammasome is also involved in caspase-1 activation. To this end, LX-2 cells were infected in the presence of A151, the oligodeoxinucleotide sequence that inhibits AIM2 or the sequence control C151. Our results indicated that AIM2 inflammasome contributes to IL-1β production by *B. abortus*-infected LX-2 cells, since the secretion of IL-1β was significantly inhibited when cells were treated with A151 with respect to untreated cells or cells treated with C151 (Figure 1C). When infection experiments were performed in the presence of A151 and glyburide administered in conjunction, the production of IL-1β was completely abrogated (Figure 1D). Taken together, these results indicated that NLRP3 and AIM2 inflammasomes are involved in the secretion of IL-1β by *B. abortus*-infected LX-2 cells.

### *B. abortus* Infection Induces ASC, NLRP3, AIM2, and Caspase-1 mRNA Expression and ASC Speck Formation in LX-2 Cells

**Figure 2:**
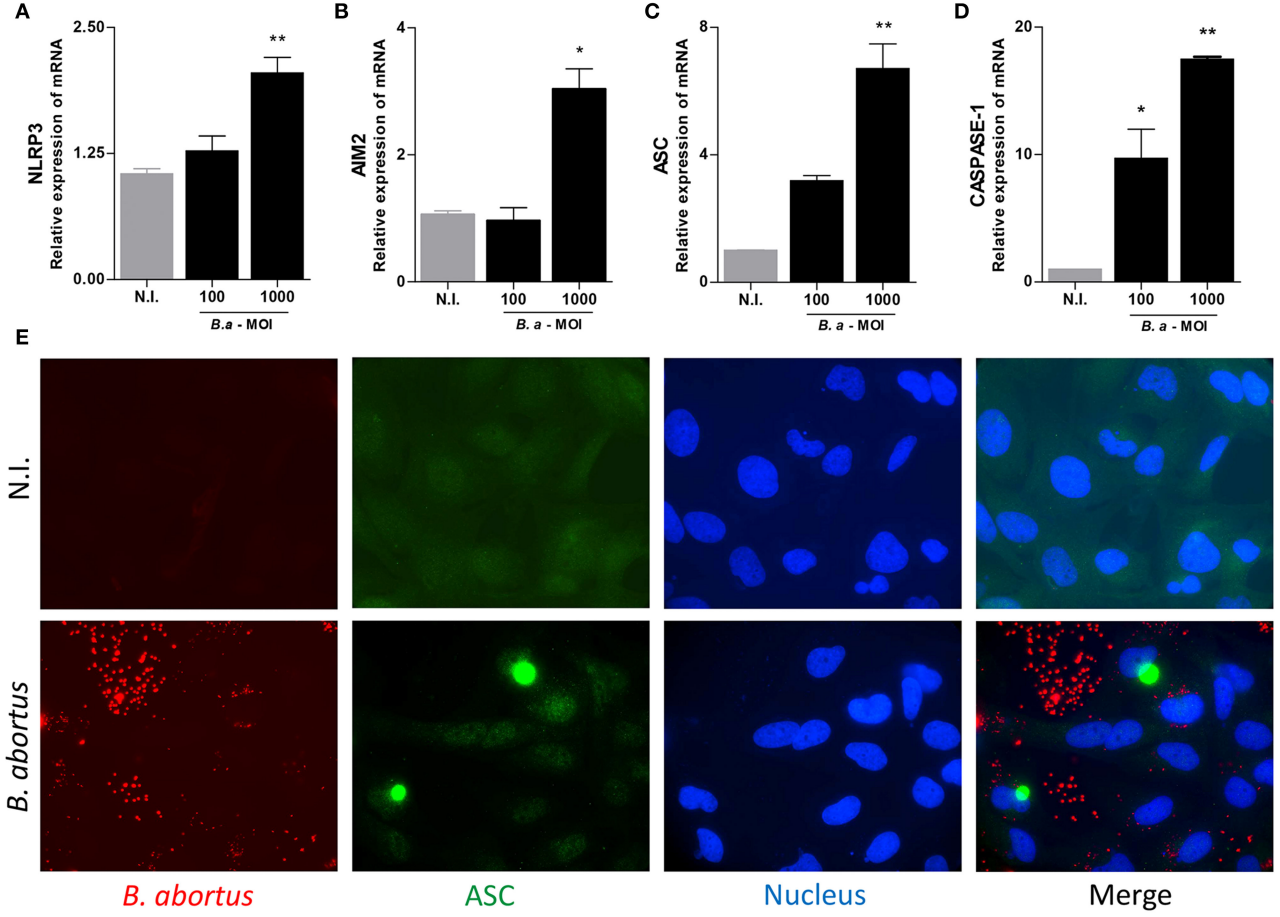
*B. abortus* induces inflammasome in LX-2 cells. LX-2 cells were infected with *B. abortus* at MOI of 100 and 1000; at 24 h, post-infection levels of NLRP3 **(A)**, AIM2 **(B)**, ASC **(C)**, Caspase 1 **(D)**, and IL-1β **(E)** were determined by RT-qPCR. LX-2 cells were infected with Ds-Red *B. abortus* and ASC speck were revealed by immunofluorescence with a specific antibody labeled with Alexa 488. Nuclei were stained with DAPI. Data are given as the means ± SD from at least three individual experiments. ^*^P < 0.05; ^**^P < 0.01 vs. non-infected cells (N.I.).

The basal AIM2 expression was sufficient to initiate inflammasome activation (20), but NLRP3 upregulation is necessary to initiate the activation of inflammasome (21, 22). Then, experiments were conducted to determine whether expression of inflammasome components could be upregulated during *B. abortus* infection. To this end, we determine the mRNA transcription of ASC, NLRP3, AIM2, and caspase-1 by RT-qPCR. Our results indicated that *B. abortus* infection induces an increase in ASC, NLRP3, AIM2, and caspase-1 mRNA transcription in LX-2 cells (Figures 2A–D). Most inflammasomes require oligomerization of ASC and thus the presence of ASC specks is a direct evidence of inflammasome activation. After infection with *B. abortus*, the formation of ASC specks was detected using specific antibodies by a fluorescence microscope. ASC specks were formed in *B. abortus*-infected LX-2 cells, but these structures were not detectable in non-infected cells. This indicates that *B. abortus* infection induces inflammasome assembly and consequently ASC speck formation (Figure 2E).

### *Brucella* DNA Induces IL-1β Secretion in LX-2 Cells

**Figure 3:**
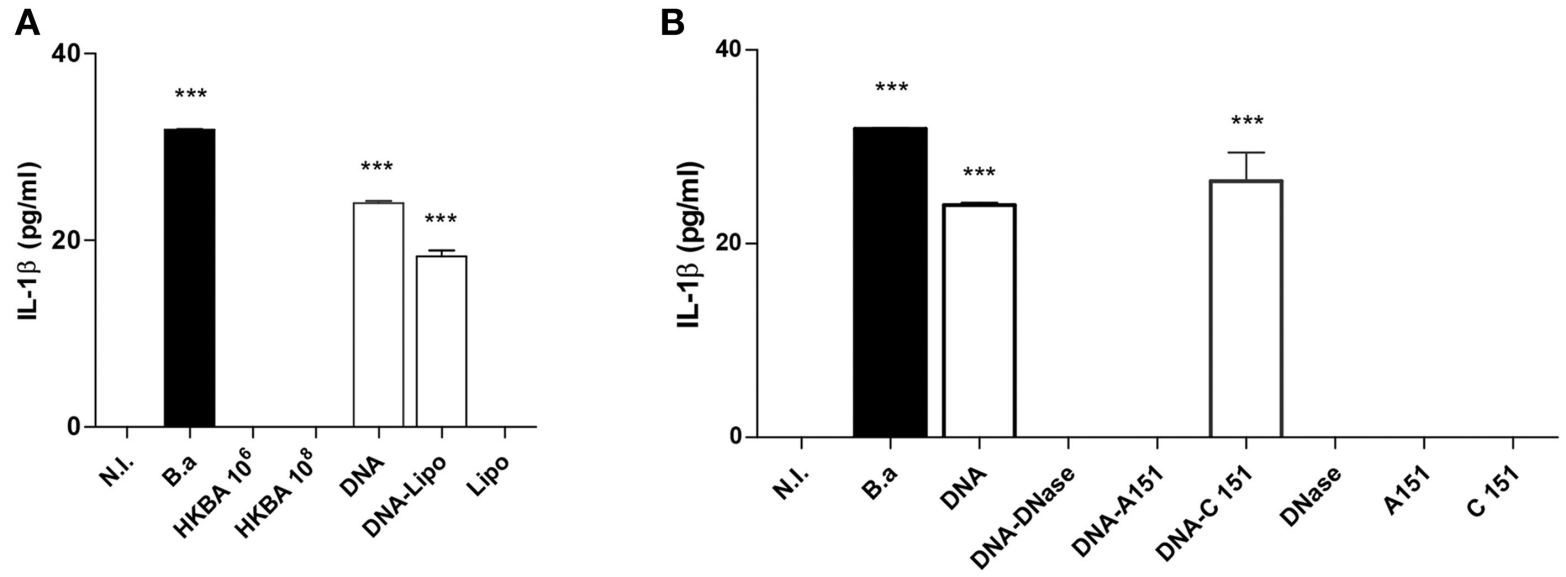
DNA from *B. abortus* induces IL-1β. **(A)** LX-2 cells were infected with *B. abortus* (*B.a*) at MOI 1000 or treated with heat-killed *B. abortus* (HKBA) (1 × 10^6^ and 1 × 10^8^ bacteria/ml), 2 μg/ml of DNA, DNA and lipofectamine (DNA + Lipo), and lipofectamine alone as control (Lipo); IL-1β secretion was measured in culture supernatant after 24 h by ELISA. Determination of IL-1β in culture supernatants from LX-2 cells treated with DNA, DNA and A151, DNA and C151 as control, DNA treated with DNAse I, and DNAse I alone as control **(B)**. Data are given as the means ± SD from at least three individual experiments. ^***^P < 0.001 vs. non-infected cells (N.I.).

IL-1β secretion was dependent on bacteria viability since stimulation of LX-2 cells with heat-killed *B. abortus* (HKBA) was unable to induce IL-1β (Figure 3A).

It has been recently demonstrated that *Brucella* DNA is involved in IL-1β secretion via activation of caspase-1 through the AIM2 inflammasome in macrophages and dendritic cells (6, 7). Therefore, we decided to test whether *Brucella* genomic DNA could be the putative ligand for AIM2 in the context of the inflammasome activation in LX-2 cells. To this end, *Brucella* DNA was transfected into LX-2 cells using lipofectamine or added to the culture medium to determine IL-1β secretion. *Brucella* DNA induced IL-1β secretion by LX-2 cells when it was added to the culture medium and also in transfected cells (Figure 3A). To determine if AIM2 inflammasome is involved in the secretion of IL-1β induced by *Brucella* DNA, experiments were performed in the presence of A151, the oligodeoxinucleotide that inhibits AIM2 or its oligodeoxinucleotide control C151. AIM2 is involved in the secretion of IL-1β induced by *Brucella* DNA, since A151 abrogated its secretion (Figure 3B). Additionally, DNase I treatment significantly reduced or abrogated *Brucella* DNA-induced IL-1β secretion (Figure 3B), demonstrating that bacterial DNA participates or is a major agonist that activates the inflammasome.

### PAMPs Associated to Inflammasome Activation

**Figure 4:**
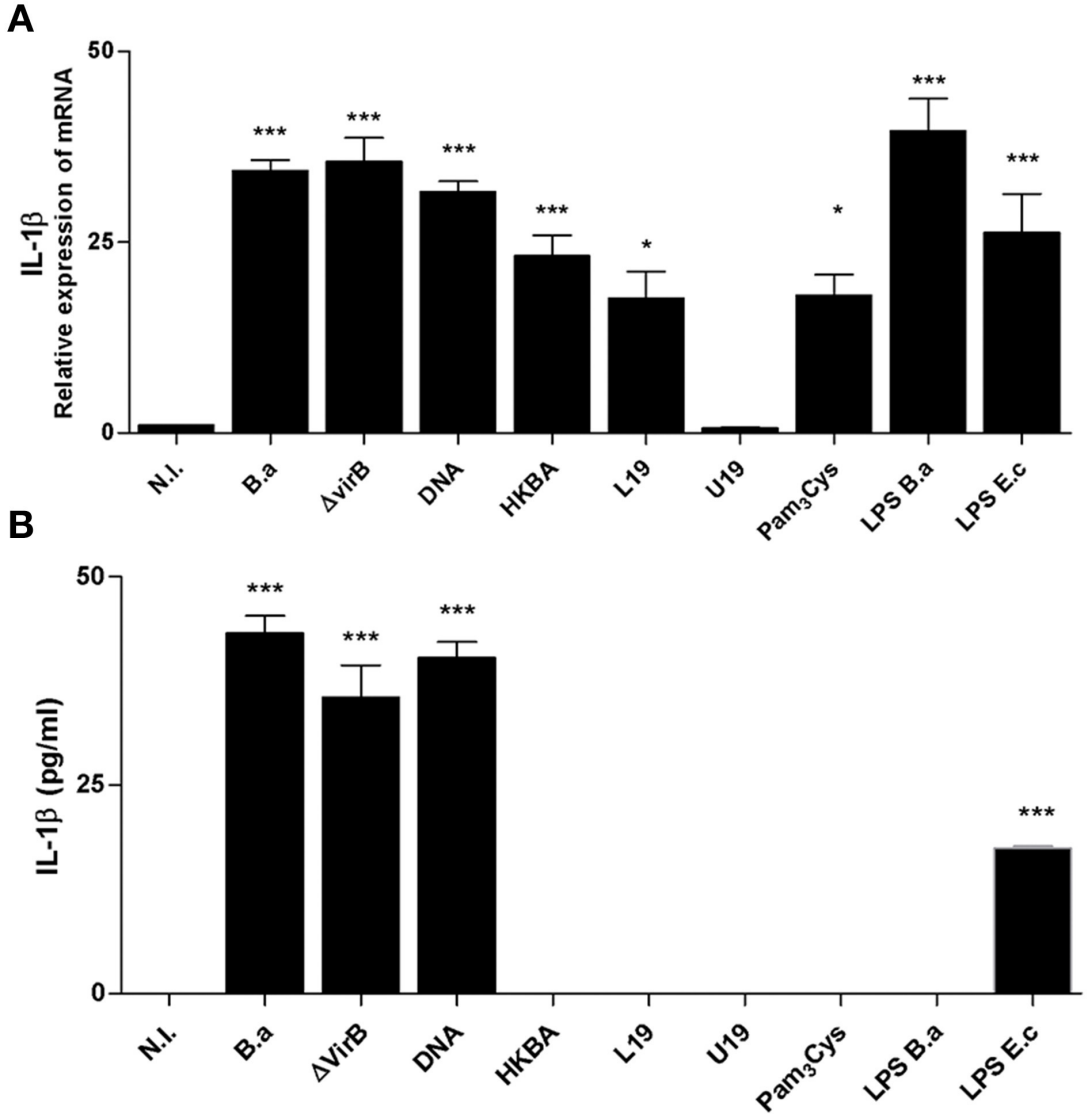
L-Omp19 induces mRNA of IL-1β. **(A)** LX-2 cells were infected with *B. abortus* (*B.a*) and its *virB10* isogenic mutant (Δ*vir*B10) at MOI 1000, or incubated with DNA (2 μg/ml), heat-killed *B. abortus* (HKBA) (1 × 10^6^ and 1 × 10^8^ bacteria/ml), L-Omp19 (1,000 ng/ml), U-Omp19 (1,000 ng/ml), *B. abortus* LPS (1,000 ng/ml), Pam_3_Cys (50 ng/ml), or *E. coli* LPS (100 ng/ml). Determination of IL-1β mRNA by RT-qPCR **(A)** and IL-1β secretion by ELISA **(B)**. Data are given as the means ± SD from at least three individual experiments. ^*^P < 0.05; ^***^P < 0.001 vs. non-infected cells (N.I.).

For the production of IL-1β, PAMPs via TLRs and NLRs function in concert. PAMPs induce the expression of the precursor form of this cytokine (pro-IL-1β), and NLR-dependent CASP-1 activation induces its proteolytic processing and release (5). Hence, to assess the role of *Brucella* PAMPs, LX-2 cells were stimulated with HKBA and mRNA expression of IL-1β was determined by RT-qPCR. Our results indicated that HKBA was able to induce IL-1β expression at the mRNA level (Figure 4A) without IL-1β release, which demonstrated that viability is crucial for IL-1β protein production.

Previous observations indicated that LPS and lipoproteins from *B. abortus* are crucial for inflammatory responses induced by *B. abortus* in different models *in vivo* and *in vitro* (16, 23–26). Cells were then incubated with LPS from *B. abortus* and lipidated Omp19 (L-Omp19) as a *Brucella* lipoprotein model, and the expression of IL-1β mRNA was determined by RT-qPCR. Our results indicated that L-Omp19 and LPS induce an increase in IL-1β mRNA expression in LX-2 cells. IL-1β mRNA expression induced by Omp19 was dependent on the lipid moiety of the molecule because unlipidated Omp19 (U-Omp19) did not induce IL-1β mRNA expression. The requirement for lipidation was further supported by the fact that Pam_3_Cys, a lipohexapeptide with an irrelevant peptide sequence, also induced the production of mRNA of IL-1β (Figure 4A). As expected, the presence of IL-1β at the protein level was not detected in supernatants of cultures of LX-2 cells treated with LPS or L-Omp19 (Figure 4B). Taken together, these results indicated that *B. abortus* lipoproteins and LPS induce mRNA of IL-1β in LX-2 cells.

### The Inflammasome Pathway Is Involved in the Profibrogenic Response of LX-2 Cells Upon *B. abortus* Infection

**Figure 5:**
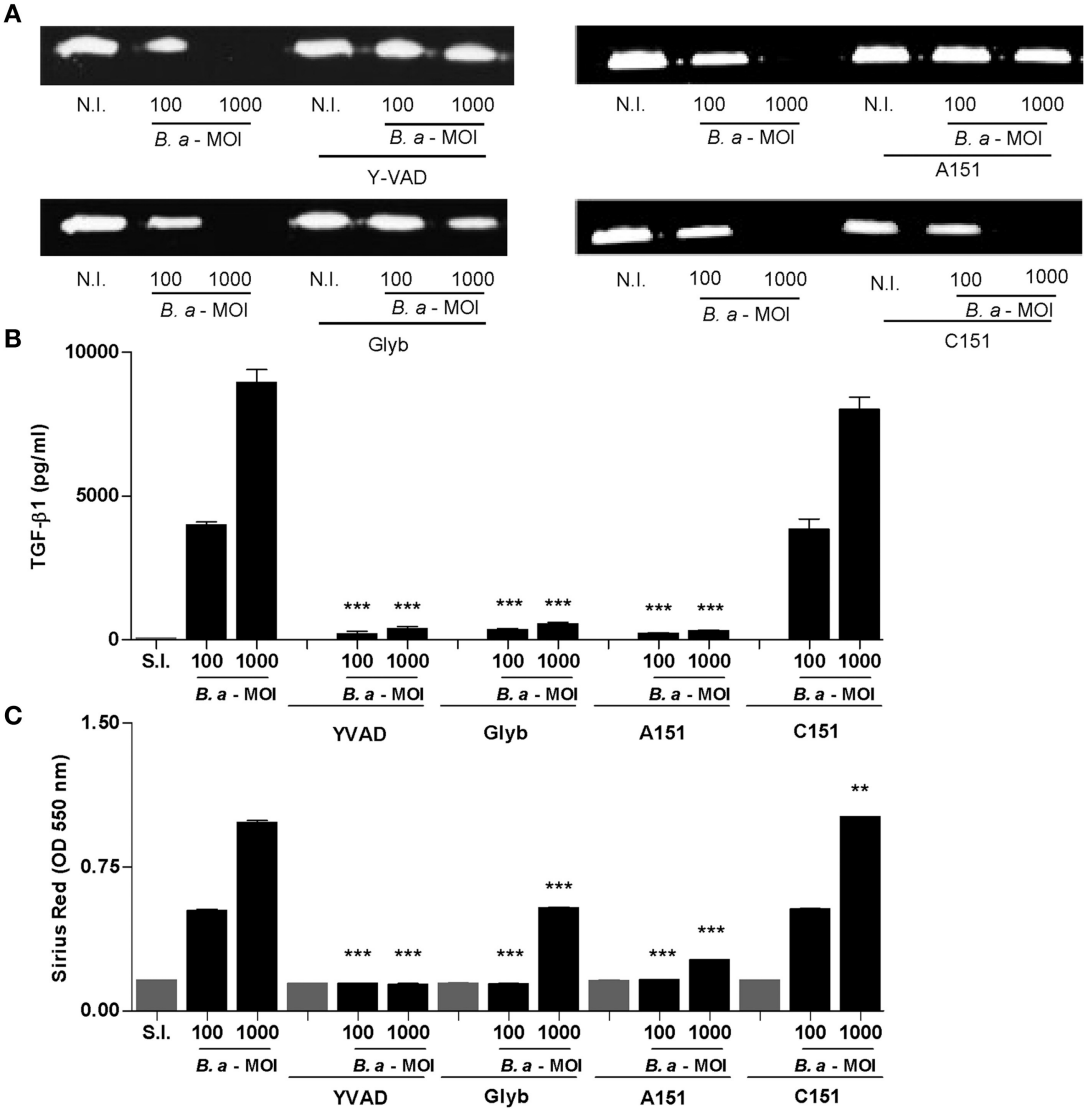
Inflammasome is involved in profibrogienic response of LX-2 cells. LX-2 cells were infected with *B. abortus* at MOI of 100 and 1000 in the presence or not of Z-VAD-FMK (ZVAD), Y-VAD-FMK (YVAD), Bay 11-7082 (Bay), and glybenclamide (Glyb). At 24 h after infection, supernatants were harvested to analyze MMP-9 production by zymography **(A)** and TGF-β1 secretion by ELISA **(B)**. Quantification of collagen deposition was revealed by Sirius red staining by OD readings at 550 nm at 10 days post-infection **(C)**. Data are given as the means ± SD of duplicates. ^**^P < 0.01; ^***^P < 0.001 vs. non-infected cells (N.I.).

It has been demonstrated that inflammasome activation has a variety of functional consequences for HSCs, including enhanced collagen 1 and TGF-β expression (4). Previously, we have demonstrated that upon infection of LX-2 cells, *B. abortus* inhibits MMP-9 secretion and induces concomitant collagen and TGF-β1 secretion (15). Therefore, experiments were conducted to determine the role of the inflammasome in the induction of a fibrogenic phenotype in LX-2 cells during *B. abortus* infection. To this end, the levels of secretion of MMP-9, TGF-β, and collagen deposition were determined in LX-2 cells infected with *B. abortus* in the presence of different inflammasome inhibitors. When infection experiments were performed in the presence of YVAD, glyburide, or A151, the effect of *B. abortus* infection on MMP-9 expression, collagen deposition, and TGF-β secretion on LX-2 cells was partially reversed with respect to untreated infected cells (Figure 5). These results indicated that NLRP3 and AIM2 inflammasome are involved in the induction of profibrogenic phenotype in LX-2 cells.

### IL-1β Is Involved in the Induction of a Profibrogenic Phenotype

**Figure 6:**
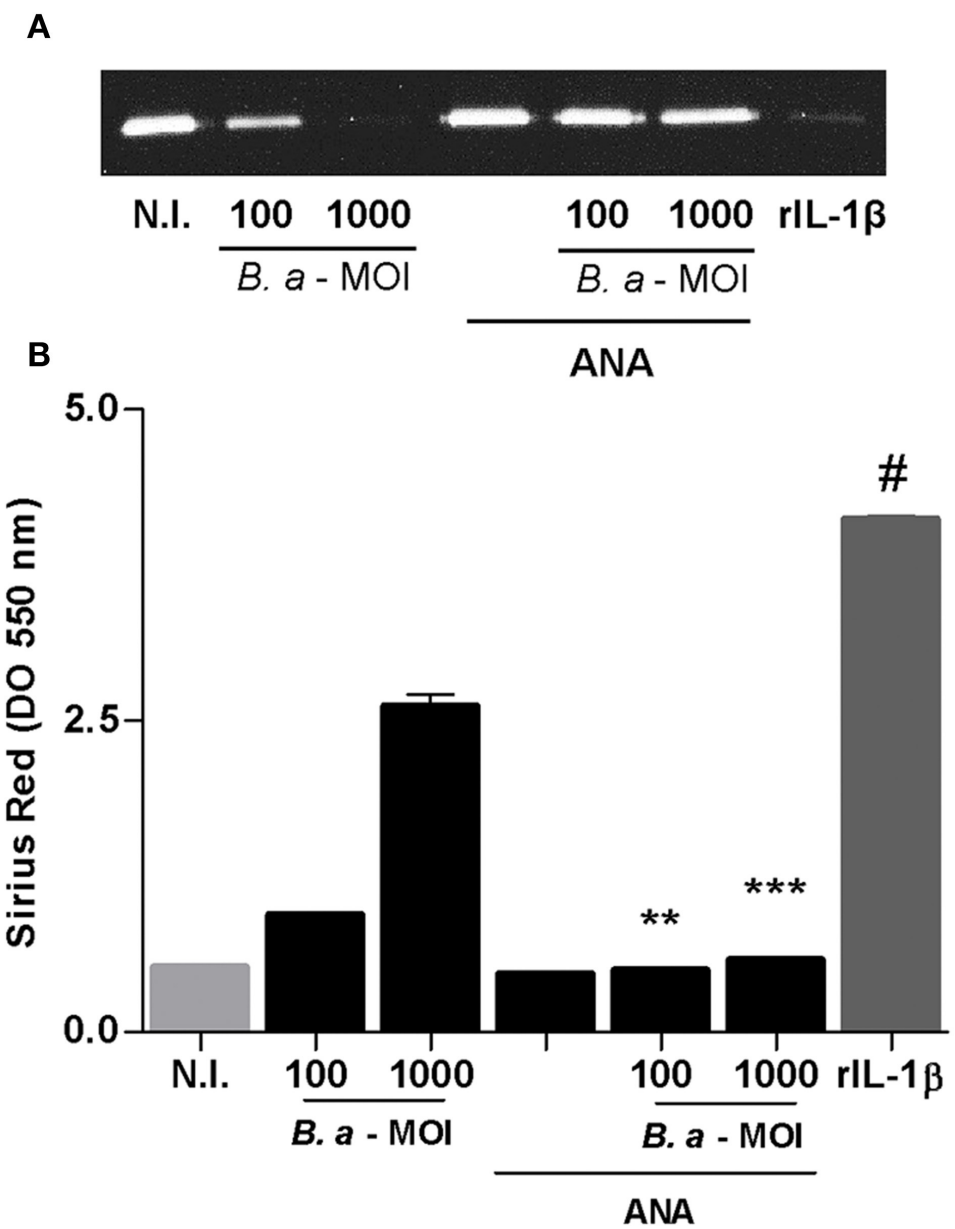
IL-1β is involved in the induction of a profibrogenic phenotype. LX-2 cells were infected with *B. abortus* at MOI of 100 and 1000 in the presence or not of ANAKINRA (ANA). Determination of MMP-9 at 24 h post-infection in culture supernatants by zymography **(A)**. Recombinant human IL-1-β, (rIL-1β) (50 ng/ml) was used as a positive control. Quantification of collagen deposition was revealed by Sirius red staining by OD readings at 550 nm at 10 days post-infection **(B)**. Data are given as the means ± SD of duplicates. ^**^P < 0.01; ^***^P < 0.001 vs. untreated cells. ^#^*P* < 0.001 vs. non-infected cells (N.I.).

Caspase-1 is required not only for IL-1β secretion but also for IL-18 secretion. To determine the role of IL-1β in the induction of a fibrotic phenotype, infection experiments were performed in the presence of the inhibitor of IL-1 receptor, the natural antagonist IL-1Ra (ANAKINRA). As shown in Figure 6, ANAKINRA abrogated the ability of *B. abortus* to inhibit MMP-9 and to induce collagen deposition. These results indicate that IL-1β could be a key cytokine during inflammasome activation involved in the fibrogenic phenotype triggered by *B. abortus* infection.

### NLRP3 and AIM2 Influence Liver Fibrosis in Livers From *B. abortus*-Infected Mice

**Figure 7:**
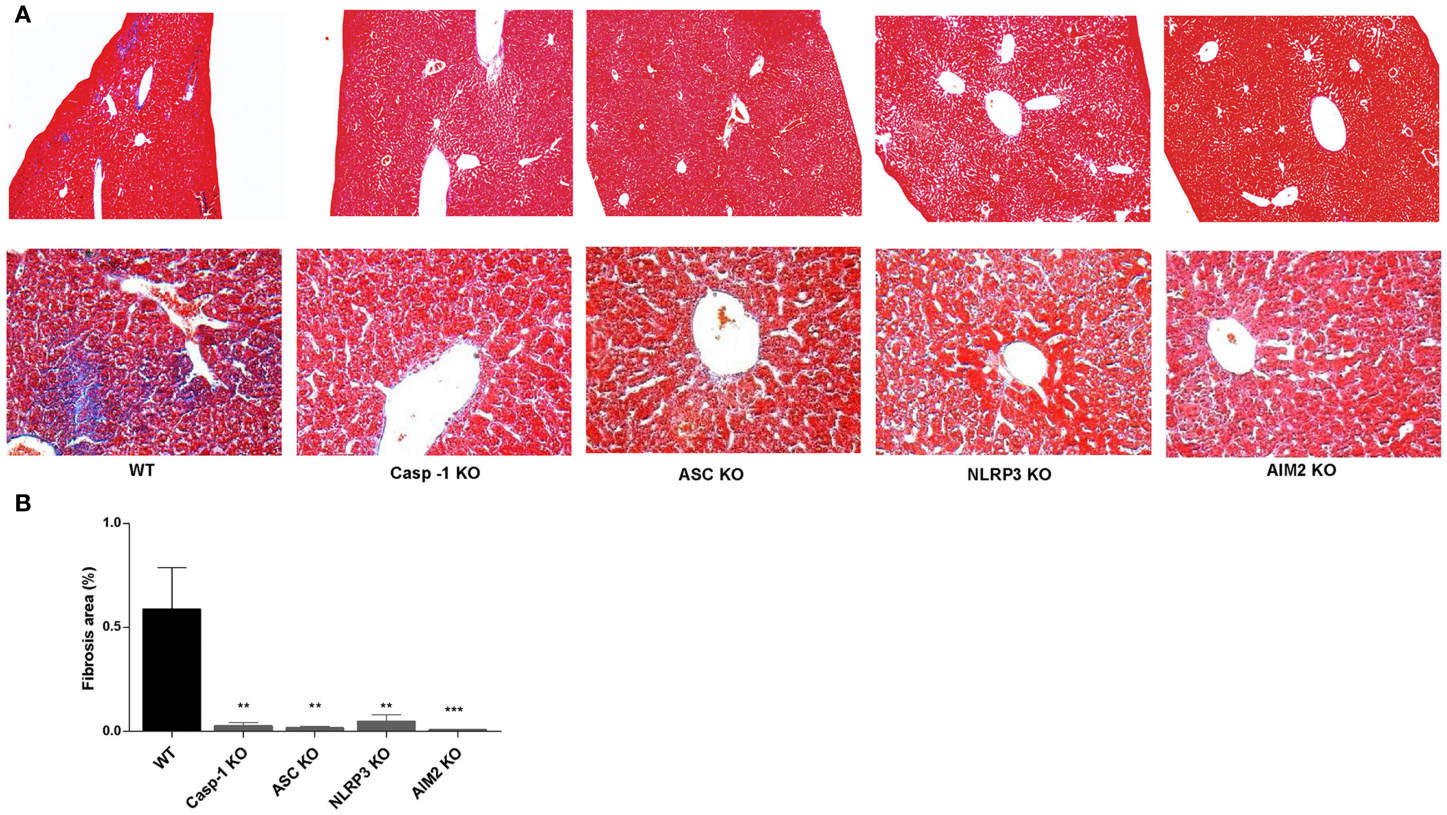
NLRP3 and AIM2 influence liver fibrosis in livers from *B. abortus*-infected mice. Representative photomicrographs of liver sections from control mice, *B. abortus-*infected mice WT, Casp-1, ASC, NLRP3, and AM2 KO mice (n5) stained with Masson's trichrome staining. The top panels show images taken at the original magnification (×100), and the bottom panels show a detail of collagen patches (magnification, ×400) **(A)**. Collagen-positive areas were quantified using Image Pro-Plus 6.0 software **(B)**. Data are given as the means ± SD from at least three individual experiments. ^**^P < 0.01; ^***^P < 0.001 vs. wild type (WT).

Finally, to verify the *in vivo* significance of our hypothesis, Casp-1, ASC, NLRP3, and AIM2 KO mice and WT mice, as control, were infected with *B. abortus*, and 4 weeks later, animals were sacrificed to determine the role of inflammasome in the liver fibrosis. Accordingly with our previous results, Masson's trichrome staining revealed the presence of fibrotic patch in livers from *B. abortus*-infected mice with respect to uninfected control (9, 15). In contrast, Casp-1, ASC, NLRP3, and AIM2 KO animals presented a significant reduction in the fibrotic patch (Figure 7). No fibrotic patch was observed in mice inoculated with saline (data not shown). These results indicated that inflammasomes NLRP2 and AIM2 play a key role in the modulation of fibrosis during *B. abortus* infection.

## Discussion

The liver is frequently affected in patients with active brucellosis, as revealed by the presence of histopathology lesions, such as granulomas, inflammatory infiltrates, and necrosis of liver parenchyma (27, 28).

Fibrosis is an intrinsic response to chronic persistent liver injury that results in a wound-healing process to mitigate the damage, but can also lead to scar formation. Inflammasome activation may play an important role in this process (19).

In the present study, we demonstrated that *B. abortus* infection activates the inflammasome with concomitant secretion of IL-1β in HSC leading to upregulation of a profibrogenic phenotype.

Inflammasomes have emerged as critical signaling molecules of innate immune system involved in liver fibrosis. Inflammasomes are intracellular multiprotein complexes that act as regulators of inflammation and cell destiny. They respond to several danger signals by activating caspase-1 by the release of proinflammatory cytokines IL-1β and IL-18 (29). In particular, the NLRP3 inflammasome has been frequently implicated in the pathogenesis of chronic inflammatory liver diseases that causes liver fibrosis (30).

HSCs are the main cells involved in extracellular matrix deposition during liver fibrosis (31). In this process, the NLRP3 inflammasome has been involved in the functional changes in HSCs, including upregulation of the expression of collagen and TGF-β (4, 32), in findings that were confirmed by performing the knocking in NLRP3 (33).

Previous studies performed in dendritic cells, macrophages, and glial cells indicate that *Brucella* is sensed by ASC-dependent inflammasomes, mainly NLRP3 and AIM2, that induce caspase-1 activation with pro-inflammatory response (6, 8, 34, 35). Accordingly, in *Brucella*-infected HSCs, the secretion of IL-1β depends on NLRP3 and AIM2 inflammasomes. In this context, the participation of PAMPs in the activation of the inflammasome and the secretion of IL-1β must be discussed. The activation and release of IL-1β requires two distinct signals. The first signal can be triggered by various pathogen-associated molecular patterns via TLR activation, which induces the synthesis of pro–IL-1β. The second signal is provided by the activation of the inflammasome and caspase-1 leading to IL-1β processing. During *B. abortus* infection, the induction of IL-1β at mRNA level was independent of bacterial viability and induced at least by two structural bacterial components, including lipoproteins and LPS. However, the second signal requires bacterial viability and the presence of a functional T4SS and *B. abortus* DNA. The T4SS is encoded by *vir*B genes that play a main role in *Brucella* intracellular replication (17), and it has also been involved in the immune response to *Brucella* infection (7, 18). Bacteria utilize the T4SS to deliver effectors to eukaryotic cells. In LX-2 cells, *B. abortus vir*B10 mutant was unable to induce IL-1β secretion, and it could suggest that *Brucella* T4SS is involved in the transport of effector molecules that act via NLRP3 and/or AIM2 to activate inflammasomes. This is in agreement with our other finding in which we demonstrate that HKBA does not induce the secretion of IL-1β by LX-2 cells.

*In vitro* studies have shown that IL-1β promotes the proliferation and myofibroblast transdifferentiation of HSCs with substantial increased levels of their fibrogenic markers (36–38). Activated caspase-1 could also cleave pro-IL-18 into its active form IL-18, and this cytokine has also been involved in fibrosis induction (38, 39). In a murine model of non-alcoholic steatohepatitis, fibrosis was not reversed by IL-1Ra treatment, indicating that other regulators of NLRP3 inflammasome are involved in fibrogenesis promotion (33). However, our experiments performed in *B. abortus*-infected HSCs in the presence of ANAKINRA, a version of the human interleukin-1 receptor antagonist, indicated that IL-1β secreted by HSCs has a main role in the induction of TGF-β with concomitant collagen deposition and inhibition of MMP-9 secretion. This role of IL-1β in the myofibroblast transdifferentiation with concomitant fibrosis is not exclusive for HSCs; accordingly, it has been previously described in endothelial and epithelial cell transdifferentiation and fibrosis (40–42).

These results and previous findings suggest that the interaction of *Brucella* with innate immunity *in vivo* may result in an increase of inflammatory response that results in liver fibrosis. Inflammasomes would dictate this fibrotic phenotype. Consequently, *B. abortus* infection induces fibrosis in mice that was reduced in mice lacking AIM2 or NLRP3.

Together, these results indicated that upon infection of HSCs, *B. abortus* triggers AIM2 and NLRP3 inflammasome activation with concomitant IL-1β secretion in a mechanism that is dependent on a functional T4SS and DNA. This IL-1β is implicated in the induction of fibrotic phenotype in HSC.

## Data Availability Statement

All datasets generated for this study are included in the article/supplementary material.

## Ethics Statement

This animal study was reviewed and approved by CICUAL-Facultad de Medicina, Universidad de Buenos Aires.

## Author Contributions

MD conceived and designed the experiments, supervised experiments, interpreted the data, and wrote the manuscript. PA, MG, and AP performed the experiments. PA analyzed the data and wrote sections of the manuscript. SO, JQ, and GG supported the work with key suggestions and helped with data interpretation. All authors reviewed the manuscript.

### Conflict of Interest

The authors declare that the research was conducted in the absence of any commercial or financial relationships that could be construed as a potential conflict of interest.
